# Failures in the Management of Elderly Women with Breast Cancer

**DOI:** 10.1371/journal.pmed.0030092

**Published:** 2006-01-17

Breast cancer is one of the highest-profile diseases in women in developed countries. Although the risk for women younger than 30 years is minimal, this risk increases with age. One-third of all breast cancer patients in Sweden, for example, are 70 years or older at diagnosis. Despite these statistics, few breast cancer trials take these older women into account. Considering that nowadays a 70-year-old woman can expect to live for at least another 12–16 years, this is a serious gap in clinical knowledge, not least because in older women breast cancer is more likely to be present with other diseases, and doctors need to know whether cancer treatment will affect or increase the risk for these diseases.

In 1992, guidelines were issued to the Uppsala/Örebro region in Sweden (with a population of 1.9 million) that all women with breast cancer should be able to receive equal treatment. At the same time, a breast cancer register was set up to record details about patients in the region, to ensure that the guidelines were being followed. Sonja Eaker and colleagues set out to assess data from the register to see whether women of all ages were receiving equal cancer treatment.

They compared the 5-year relative survival for 9,059 women with breast cancer aged 50–84 years. They divided them into two age groups: 50–69 years, and 70–84 years. They also categorized the women according to the stage of breast cancer. They looked at differences between the proliferative ability of breast cancer cells, estrogen receptor status, the number of lymph nodes examined, and lymph node involvement. The researchers also compared types of treatment—i.e., surgical, oncological (radiotherapy, chemotherapy, or hormonal)—and the type of clinic the patients were treated in.

They found that women aged 70–84 years had up to a 13% lower chance of surviving breast cancer than those aged 50–69 years. Records for older women tended to have less information on their disease, and these women were more likely to have unknown proliferation and estrogen receptor status.

Older women were less likely to have their cancer detected by mammography screening and to have the stage of disease identified, and they had larger tumors. They also had fewer lymph nodes examined, and had radiotherapy and chemotherapy less often than younger patients. Current guidelines are vague about the use of chemotherapy in older women, since studies have included only a few older women so far, but this did not explain why these women received radiotherapy less often. Older women were also less likely to be offered breast-conserving surgery, but they were more likely to be given hormone treatment such as tamoxifen even if the tumors did not show sign of hormone sensitivity. The researchers suggest that this could be because since chemotherapy tends to be not recommended for older women, perhaps clinicians believed that tamoxifen could be an alternative.

The researchers admit that one drawback of their study is that there was little information on the other diseases that older women had, which might explain why they were offered treatment less often than younger patients.

However, the fact remains that in Sweden, women older than 70 years are offered mammography screening much less often than younger women—despite accounting for one-third of all breast cancer cases in the country—and those older than 74 years are not screened at all.

Eaker and coworkers' findings indicate that older women are urgently in need of better treatment for breast cancer and guidelines that are more appropriate to their age group. Developed countries, faced with an increasingly aging population, cannot afford to neglect the elderly.

## 

**Figure:**
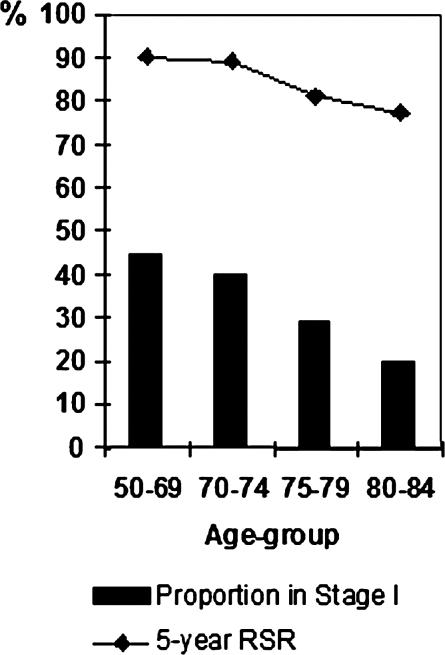
The proportion of women diagnosed in stage I breast cancer and the 5-year cumulative relative survival ratio differ by age group.

